# Complete Genome Sequence of *Klebsiella oxytoca* Strain JKo3

**DOI:** 10.1128/genomeA.01221-16

**Published:** 2016-11-03

**Authors:** Tadayuki Iwase, Yoshitoshi Ogura, Tetsuya Hayashi, Yoshimitsu Mizunoe

**Affiliations:** a Department of Bacteriology, The Jikei University School of Medicine, Nishi-Shinbashi, Minato-ku, Tokyo, Japan; b Department of Bacteriology, Faculty of Medical Sciences, Kyushu University, Maidashi, Higashi-ku, Fukuoka, Japan

## Abstract

*Klebsiella oxytoca* can be either pathogenic or beneficial, depending on conditions. These opposing characteristics have not been fully elucidated. Here, we report the complete sequence of the *K. oxytoca* JKo3 genome, consisting of a single circular chromosome of 5,943,791 bp and four plasmids.

## GENOME ANNOUNCEMENT

The genus *Klebsiella* belongs to the family *Enterobacteriaceae* and contains nonmotile rod-shaped Gram-negative bacteria. *Klebsiella oxytoca* is a free-living bacterium that can be isolated from various plants and animals, including humans. The species can be either pathogenic or beneficial depending on conditions, because it occasionally causes colitis in humans but also promotes the growth of plants and insects as an endophytic diazotroph (1–9). These bilateral characteristics of the species require further elucidation. To provide a genetic basis for such analyses, we determined the genome sequence of strain JKo3, which is a clinical isolate in the strain collection at the Jikei University School of Medicine in Japan (10).

The sequence of JKo3 was determined using the 454 GS FLX Titanium system. Using the GS Assembler software version 2.6, a total of 466,772 single-end reads (162 Mb in total) and 141,520 8-kb paired-end reads (44 Mb) were assembled into eight scaffolds containing 92 gaps. Finishing was first performed by *in silico* analysis using GenoFinisher (11), and then remaining gaps were closed by sequencing gap-spanning PCR products using an ABI3130xl DNA sequencer (Applied Biosystems). To correct sequence errors, a paired-end library of JKo3 was constructed using the TruSeq DNA sample prep kit (Illumina) and sequenced by Illumina MiSeq (2 × 150 bp). Mapping of the obtained 1,998,167 reads to the JKo3 genome sequence and single-nucleotide polymorphism (SNP) calling were performed using BWA (12) and SAMtools (13), respectively. A total of 20 insertions/deletions were corrected by the resequencing. Gene identification and annotation were conducted by the Microbial Genome Annotation Pipeline (MiGAP [http://www.migap.org]).

The chromosome of JKo3 is 5,943,791 bp in size. It contains eight copies of rRNA operons, 83 tRNA genes, and 5,647 protein-coding genes. *K. oxytoca* is known to contain a *nif* gene cluster, which is a model system for studying nitrogen fixation (6). The *nif* gene cluster of JKo3 consisted of 20 genes and was found in a 23-kb chromosome region. The number and orientation of genes were consistent with a previous report (10, 14). In addition, JKo3 has one small plasmid (plasmid 1 [3,897 bp], containing four protein-coding genes), and three large plasmids (plasmid 2 [80,479 bp], plasmid 3 [104,259 bp], and plasmid 4 [208,080 bp], containing 50, 91, and 101 protein-coding genes, respectively). These plasmids could be used for the construction of a set of plasmid vectors useful in the molecular or biotechnology fields because they are compatible with each other. The complete genome sequence of *K. oxytoca* JKo3 presented here could provide useful information for investigating the beneficial and nosocomial pathogenic characteristics of *K. oxytoca*.

### Accession number(s).

This whole-genome shotgun project for JKo3 has been deposited at DDBJ under the accession numbers AP014951 (chromosome), AP014952 (plasmid 1), AP014953 (plasmid 2), AP014954 (plasmid 3), and AP014955 (plasmid 4).
